# Sex differences in ketogenic diet: are men more likely than women to lose weight?

**DOI:** 10.3389/fnut.2025.1600927

**Published:** 2025-06-04

**Authors:** Yingying Jiao, Xiuru Chen, Lu Liu, Yantong Lu, Minghuang Gao, Qi Wang, Xiaoling Chi, Yousheng Mo

**Affiliations:** ^1^Science and Technology Innovation Center, Guangzhou University of Chinese Medicine, Guangzhou, Guangdong, China; ^2^Department of Hepatology, The Second Affiliated Hospital of Guangzhou University of Chinese Medicine, Guangdong Provincial Hospital of Chinese Medicine, Guangzhou, Guangdong, China

**Keywords:** ketogenic diet, obesity, sex differences, metabolism, mechanism

## Abstract

As economy is growing, the number of patients living with obesity has rapidly increased globally. Patients with obesity or diabetes have become a serious global health issue that requires the attention and participation of society as a whole. The ketogenic diet, as an emerging nutritional therapy for improving obesity, takes into consideration the differences between sexes in genetic variation, hormonal balance, and body fat distribution. The aim is to elucidate the effectiveness of sex differences in ketogenic dieting for weight loss and to explore suitable weight loss strategies. In this review, we delve into the physiological sexual differences between men and women in terms of fat and muscle tissue and discuss the sex-specific potential mechanisms underlying the differential effects of the ketogenic diet for weight loss. Based on this foundation, we further propose brief weight loss recommendations beneficial for both men and women. It is hoped that, in this direction, the optimization of short-term or long-term clinical weight loss programs can be developed based on sexes.

## Introduction

Obesity is globally recognized as a significant public health challenge and is closely linked to diseases such as type 2 diabetes, cardiovascular disorders, and various cancers. It is also one of the main reasons for the high prevalence and mortality rates of metabolic diseases (1). As of March 2023, the “World Obesity Map” indicated that in 2020, out of the global population aged over 5, 2.6 billion people were obese or overweight. By 2035, this number is projected to exceed 4 billion, rising from 38% in 2020 to over 50% in 2035 (2). In recent years, dietary interventions for obesity have become a point of contention, with many different weight-loss methods being promoted (3). Among them, the traditional low-fat diet is widely used, but it also has its drawbacks. While it can promote the consumption of carbohydrates, this might exacerbate weight issues and encourage lipid abnormalities (4), especially in individuals with insulin resistance. In contrast to the classic low-fat, high-carbohydrate diet, the KD is a high-fat, ultra-low-carbohydrate, and moderate-protein dietary approach. Its core objective is to induce a state of ketosis, in which the body primarily relies on ketone bodies rather than glucose as its main energy source. Today, KD has gained widespread popularity as an effective weight-loss method. In a randomized controlled clinical trial where subjects were randomly assigned to a 6-month KD or a high-carb, low-fat diet under controlled variables, results showed that the KD group lost weight faster and shed more weight throughout the trial, without associated cardiovascular risks emerging within the 6 months (5). Studies suggest that the KD not only helps patients with obesity or diabetes lose weight (6) but also aids in increasing insulin sensitivity in type 2 diabetes patients and improves blood sugar control (7). In a 45-day study of very-low-energy ketogenic therapy (VLEKT) involving 21 premenopausal women and 21 men, men experienced a significantly greater weight loss than women, with a mean percentage decrease of 11.63% (11.63 ± 1.76 kg) compared to 8.95% (8.95 ± 1.65 kg) in women (8). In another two-diet period clinical intervention study, researchers found that, compared to a low-fat diet, ketogenic diet offered a distinct advantage for men in terms of weight loss, total fat loss, and trunk fat loss (despite a significantly higher energy intake) (9).

Although the KD shows significant results in weight loss, there is a noticeable sex difference in its effects. Epidemiological, physiological, and clinical therapy research has reported sex differences in the KD’s treatment of obesity (10). While social and psychological factors undoubtedly play roles in the observed discrepancies in prevalence and incidence, biological differences in heredity, gonadal hormone, such as testosterone and estrogen, and lipid metabolism might underlie these observed effects. This review synthesizes current literature discussing the mechanisms of the KD in treating obesity and the sex disparities arising from interactions between innate factors and hormones under the KD. We believe that by enhancing our understanding of the challenges in this field, we will lay the groundwork for urgently needed research, paving the way for more personalized and targeted treatments in obesity.

## Review methodology

A comprehensive literature search was conducted using databases such as PubMed, Scopus, Embase, Web of Science, and the Cochrane Library. The search strategy was tailored with specific keywords to ensure relevance; for example, in PubMed, these included (“Ketogenic Diet”[Mesh] OR “ketogenic diet” OR “low carbohydrate diet” OR “high fat diet”) AND (“Weight Loss”[Mesh] OR “weight loss” OR “body weight reduction” OR “fat loss” OR “obesity”[Mesh]) AND (“Sex Characteristics”[Mesh] OR “sex differences” OR “gender differences” OR “male” OR “female” OR “men” OR “women”), along with other similar terms and phrases relevant to the topic.

Inclusion criteria: Studies specifically addressing the effects of KD on weight loss and those involving gender differences were included. Preference was given to recent research articles, randomized controlled trials, cohort studies, case–control studies, and clinical trials, while human and animal studies, as well as *in vitro* studies, were considered if relevant to the research topic.

### Exclusion criteria

Non-English articles, reviews, editorials, and those without accessible full text were excluded. While literature reviews were generally excluded, exceptions were made if they provided substantial insights or unique perspectives not found in original research articles.

Potential articles identified through this process were initially checked for duplicates by two reviewers (Y. J. and Y. M.). The applicability of titles and abstracts was then screened by three reviewers (L. L., Y. L., and M. G.), and full-text reviews were conducted by three reviewers (Y. J., Q. W., and X. C.) based on the results of this stage. The resulting article set was thoroughly examined by all reviewers, with important findings extracted, summarized, and analyzed.

## The background of the KD

### The history and definition of the ketogenic diet

Originating from a dietary strategy rich in fats, moderate in protein, and scarce in carbohydrates, the KD has a legacy that dates back to the time of Hippocrates (460–370 BCE) (11). Initially, its potential was harnessed in the realm of medicine, particularly as a remedy for refractory epilepsy. The pioneering utilization of the KD was in 1911 when two Parisian physicians, Gulep and Marie, recognized its therapeutic advantages for epilepsy (12). Their observations highlighted a reduced severity of seizures in both children and adults, though the specifics remained largely uncharted. By the 1920s, the KD was championed by Dr. Hugh Conklin, bringing relief to countless young epilepsy patients (13). The diet became a cornerstone treatment during the 1920s and 1930s. However, with the introduction of the groundbreaking antiepileptic drug phenobarbital (Dilantin), the KD’s prominence faded, overshadowed by challenges like patient adherence.

Traditional ketogenic diets (KD) adhere to a 4:1 ratio of fat to the combined total of carbohydrates and protein (grams) (14–16). The stringent ratio requirement presented challenges with regards to adherence to the ketogenic diet, spurring subsequent researchers to refine the ratios, such as the introduction of the medium-chain triglyceride (MCT) diet, which elevates the proportion of MCTs to enhance ketone production efficiency (17). Subsequently, the modified Atkins Diet (MAD) ratio was proposed in the 1970s by American cardiologist Robert C. Atkins as a low-carbohydrate dietary regimen. MAD entails a ketogenic ratio of 0.9:1 (fat: carbs to protein), which is slightly lower than that of the classic KD, with the primary goal of increasing urinary ketones to achieve the secondary endpoint of weight loss (18). Overall, there is currently no standardized definition of the KD. Different versions of KD adhere to the same core principle: high fat and low carbohydrate intake, with approximately 50% or more of total caloric intake derived from fat (19).

### The mechanism behind the KD

In a typical diet, carbohydrates stand as the primary energy pillar, metabolizing into glucose, which further transforms into pyruvate. This pyruvate, upon oxidation, gives rise to Acetyl-CoA, an integral player in the tricarboxylic acid cycle (TCA cycle). Parallelly, fats, when metabolized, break down into glycerol and fatty acids. These fatty acids, after undergoing β-oxidation, also produce Acetyl-CoA, fueling the TCA cycle to generate energy. Under standard dietary conditions, only minimal ketones are produced, too insignificant to elicit notable metabolic reactions (20). Embracing the KD tricks the body into mimicking a fasting state, with carbohydrate scarcity leading to a significant accumulation of acetyl-CoA. This surfeit propels the liver into overdrive, churning out an excess of ketones (21), which is a term encompassing β-hydroxybutyrate (β-OHB), acetoacetate, and acetone—the by-products of fat metabolism.

The blood–brain barrier restricts energy sources for the brain mainly to glucose and ketones (22). In situations like fasting, ketones can cater to a considerable 25–75% of the brain’s energy demands (23). Therefore, the KD can maintain normal brain energy supply while keeping peripheral blood glucose levels and decreasing insulin sensitivity, thereby reducing lipogenesis and promoting fat breakdown (24–26).

**Figure 1:**
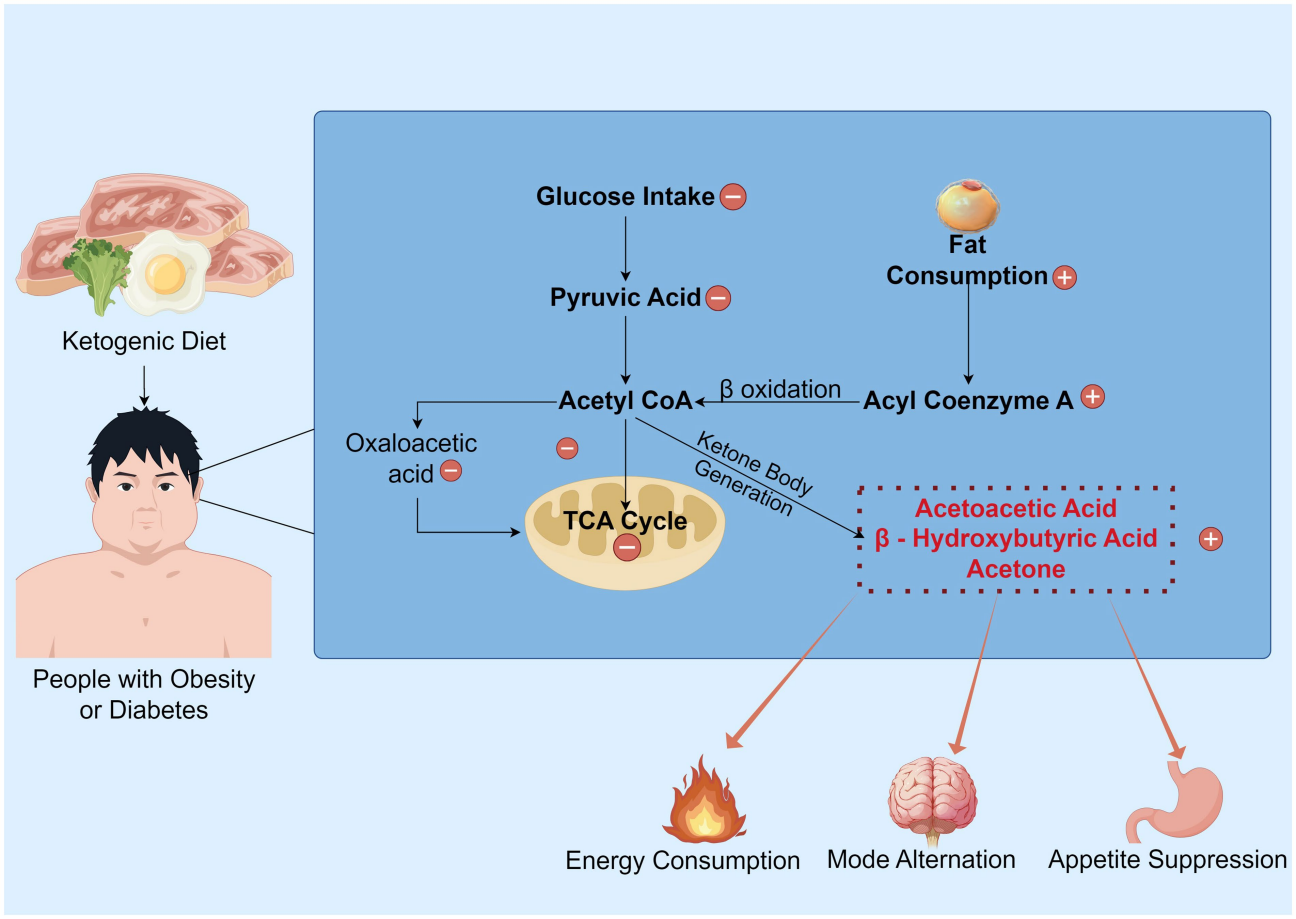
The overview of the KD’s mechanism. Under normal metabolic conditions, glucose serves as the primary energy substrate and is metabolized into pyruvate. This pyruvate is then converted to acetyl CoA, generating oxaloacetate, which enters the TCA cycle to produce ATP. However, under ketogenic dietary states, the synthesis of oxaloacetate is restricted, impeding the normal progression of the TCA cycle. Consequently, a substantial consumption of fat occurs, with processes such as fatty acid activation and β-oxidation generating acetyl-CoA, which promotes the production of ketone bodies. Subsequently, this ketogenesis process yields energy and inhibits appetite, while also shifting the brain to a “fat-fueled” energy mode. Some parts created with Figdraw.com.

In the peripheral circulation, the KD primarily promotes weight loss through several key mechanisms. First, it reduces food intake. KD suppresses appetite by increasing peptide neurotransmitters such as peptide (PYY) and glucagon-like peptide-1 (GLP-1) while decreasing levels of appetite-regulating hormones like cholecystokinin (CCK) and stomach ghrelin concentrations, thereby reducing caloric consumption (27–29). Second, it enhances the breakdown of existing visceral fat. KD reduces the storage of liver glycogen and water as well as the accumulation of visceral fat. The lipolytic effect is amplified in the ketogenic state, and the decomposed fatty acids can be further converted into ketone bodies for energy. Additionally, a high-sugar diet exacerbates inflammatory responses (30, 31), affecting the energy demands of central nervous system (CNS) neurons. A low-sugar diet significantly improves this condition (29). Finally, KD alters the structure and function of the gut microbiota, reducing the production of short-chain fatty acids (SCFAs), which in turn affects the signaling of the gut-brain axis (32). This modulation may further suppress appetite and promote weight loss (33) (Figure 1).

## Sex differences in body composition

### Adipose tissue

In humans and other mammals, there is a striking divergence in how body fat is distributed between the sexes. Men tend to accumulate fat centrally, presenting a pronounced visceral fat profile that deposits in the chest, abdomen, and buttocks, leading to an ‘apple-shaped’ physique. Conversely, premenopausal women generally store more subcutaneous fat, particularly in areas like the breasts, hips, legs, and waist, creating a ‘pear-shaped’ silhouette (34–36).

Many women fall into the category of “metabolically healthy obesity” (with higher insulin sensitivity/absence of insulin resistance; more body fat but normal amounts of abdominal fat tissue; relatively less visceral and ectopic fat, primarily accumulating subcutaneously; normal metabolic indicators such as blood lipids, blood pressure, and blood glucose; and low levels of inflammation and oxidative stress), while men are more likely to experience metabolically unhealthy obesity (37). This central fat accumulation, combined with more significant hormonal shifts, predisposes men to endocrine disruptions and increases their risk for cardiovascular diseases, insulin resistance, hypertension, and diabetes (38). On the other hand, compared to the metabolically slower subcutaneous fat, visceral fat, particularly abdominal fat, is more easily burned by the KD, resulting in significant weight loss in men.

### Muscle tissue

Engaging in muscle exercise can enhance basal metabolic rates, facilitating efficient calorie consumption. Research underscores that muscle augmentation is positively correlated with increased basal metabolic rates, expediting energy expenditure. The pronounced sex disparity in muscle development becomes evident during puberty (39); boys tend to have more pronounced muscle development than girls. In adulthood, for a given body weight, males generally possess a greater muscle mass compared to females. During endurance exercises, females might also exhibit an increased release and uptake of fatty acids (FA) in skeletal muscles (40). A study gauging ketone concentrations post-exercise revealed that female mice had serum ketone levels approximately 45% higher than their male counterparts after endurance activities. Consequently, female mice produce more exercise-induced ketones.

Human muscle fibers can be generally classified into three types: Type I, Type II and Type IIx, with the first two being the most predominant. Type I fibers are rich in mitochondria at their periphery and are proximal to the intramuscular capillaries. Consequently, they possess a strong aerobic metabolism capacity for glucose and free fatty acids (FFA), exhibit heightened insulin sensitivity (41), and are more resistant to fatigue. On the other hand, Type II fibers have a lower capillary density and mitochondrial concentration compared to Type I fibers. They rely less on aerobic metabolism and more on anaerobic energy production (42), resulting in faster contraction speeds and higher force output per contraction unit but tending to fatigue more quickly. Studies indicate that, compared to males, females are more inclined toward oxidative metabolism (43). Within the same muscle, females also have a higher proportion of Type I fibers, naturally translating to a higher capillary density (44). This, to some extent, indicates that perhaps women are more suited to aerobic exercise rather than the ketogenic diet for weight loss.

## Underlying mechanisms of sex variations in the KD weight loss

### Heredity

Although no studies have yet systematically delved into the genetic underpinnings of sex differences induced by the KD, a synthesis of the extant literature suggests that these genetic sex disparities under the KD might be intricately associated with neurotransmitter levels, individual sensitivity to varied environmental stimuli, and certain intermediate phenotypes. Specifically, the KD is found to diminish the concentrations of central nervous system neurotransmitters like norepinephrine (NE) and dopamine (DA), as well as serotonin (5-HT), subsequently influencing feeding behaviors. Catecholamines can inhibit appetite and reduce food intake and appetite through neural pathways (45). This helps to control diet and decrease the total calorie intake. Dopamine accounts for approximately 80% of the brain’s catecholamine content. Studies have found that among all food categories, carbohydrates are typically considered the most addictive, and high-carbohydrate diets stimulate the mesolimbic dopamine pathway, leading to excessive food intake and obesity (46). The ketogenic diet, to some extent, can prevent this from happening. In the non-fasting Mediterranean diet state, the brain’s primary fuel is glucose, and fluctuations in blood sugar levels trigger changes in the firing of dopamine neurons in the striatum (47). Every time you consume sweets, the brain’s reward system—the mesolimbic dopamine system—is activated (48).

In the realm of catecholamine-mediated lipolysis, variations are more salient in males than in females. For instance, NE’s lipolytic prowess in abdominal adipocytes surpasses that in gluteal adipocytes. As previously noted, men tend to accumulate fat around their abdominal organs, while women predominantly store fat subcutaneously in the buttocks and thighs. This sex-specific difference in fat distribution may be linked to the distinct effects of norepinephrine (NE) on regional adipose tissue, potentially accounting for the observed variations in the weight loss effects of the ketogenic diet (KD) between males and females (49).

Another intriguing discovery comes from a study that showcased a consistent density of α2-adrenergic receptors across various tissues. However, in female specimens, the affinity of clonidine (a targeted α2-adrenergic agonist) in abdominal adipocytes was discernibly lower—by 10–15 times—than in their gluteal counterparts. This region-specific disparity in catecholamine-induced lipolysis can be attributed to the site-specific variations in the density of β-adrenergic receptors. The modulations in the affinity of α2-adrenergic receptors in females provide, at the very least, a partial rationale for the augmented catecholamine-induced lipolytic response observed in males (50).

### Sex hormone

#### Estrogen

The differential response to the KD across sexes may, in part, be attributed to the modulatory effects of estrogen. In a study where both male and female murine models were subjected to the KD for a duration of 15 weeks, male subjects effectively maintained glycemic homeostasis and exhibited weight reduction (51). In contrast, the female cohort manifested a minor weight augmentation, coupled with delayed onset of insulin resistance and compromised glucose tolerance. Intriguingly, following oophorectomy to eradicate endogenous estrogen production, females on the KD demonstrated a reduction in adiposity and improved glycemic control, paralleling the metabolic effects observed in males.

Additionally, preliminary findings suggest that short-term adherence to the KD may induce a surge in serum cortisol concentrations to transition the body to utilizing fat as its principal energy source (52), a glucocorticoid synthesized in the adrenal cortex. Elevated cortisol can potentiate estrogenic activity. The interaction between estrogen and cortisol may enhance women’s craving for high-sugar and high-fat foods, particularly during the luteal phase (53). However, testosterone may partially counteract cortisol’s appetite-stimulating effects of men (54). Exorbitant estrogen levels in females can impede thyroid hormone synthesis, potentially inducing bidirectional interference between estrogen and the hypothalamic–pituitary–adrenal (HPA) axis. Notably, recalcitrant adipose tissues are enriched with α-adrenergic receptors. While α-receptors play an inhibitory role in lipase activity, modulating muscular energy provision during exertion and curtailing lipolysis, β-receptors stimulate lipase, facilitating muscular contractility and fostering lipid catabolism (55). Estrogen is known to amplify both the sensitivity and abundance of *α*-adrenergic receptors, attenuating the adrenergic-mediated lipolytic response in subcutaneous adipocytes (56). This physiological complexity may offer new insights into why women experience relatively greater difficulties than men in achieving fat reduction through a ketogenic diet.

#### Testosterone

Testosterone is a hormone pivotal in the metabolism of carbohydrates, fats, and proteins. Given that its concentration in males is typically about 10 times higher than in females, it is often termed the “male hormone.” Accumulating evidence suggests that testosterone orchestrates the expression of key regulatory proteins involved in glycolysis, glycogenesis, lipid, and cholesterol metabolism at the molecular level (57). Metabolic shifts driven by testosterone in adipocytes lead to a reduction in the production of free fatty acids (FFA), subsequently mitigating insulin resistance (58). Early studies demonstrated that testosterone amplifies norepinephrine (NE)-induced lipolysis in isolated adipocytes from normal male rats (59) and can enhance lipolysis by increasing the number of β-adrenergic receptors (60).

**Figure 2:**
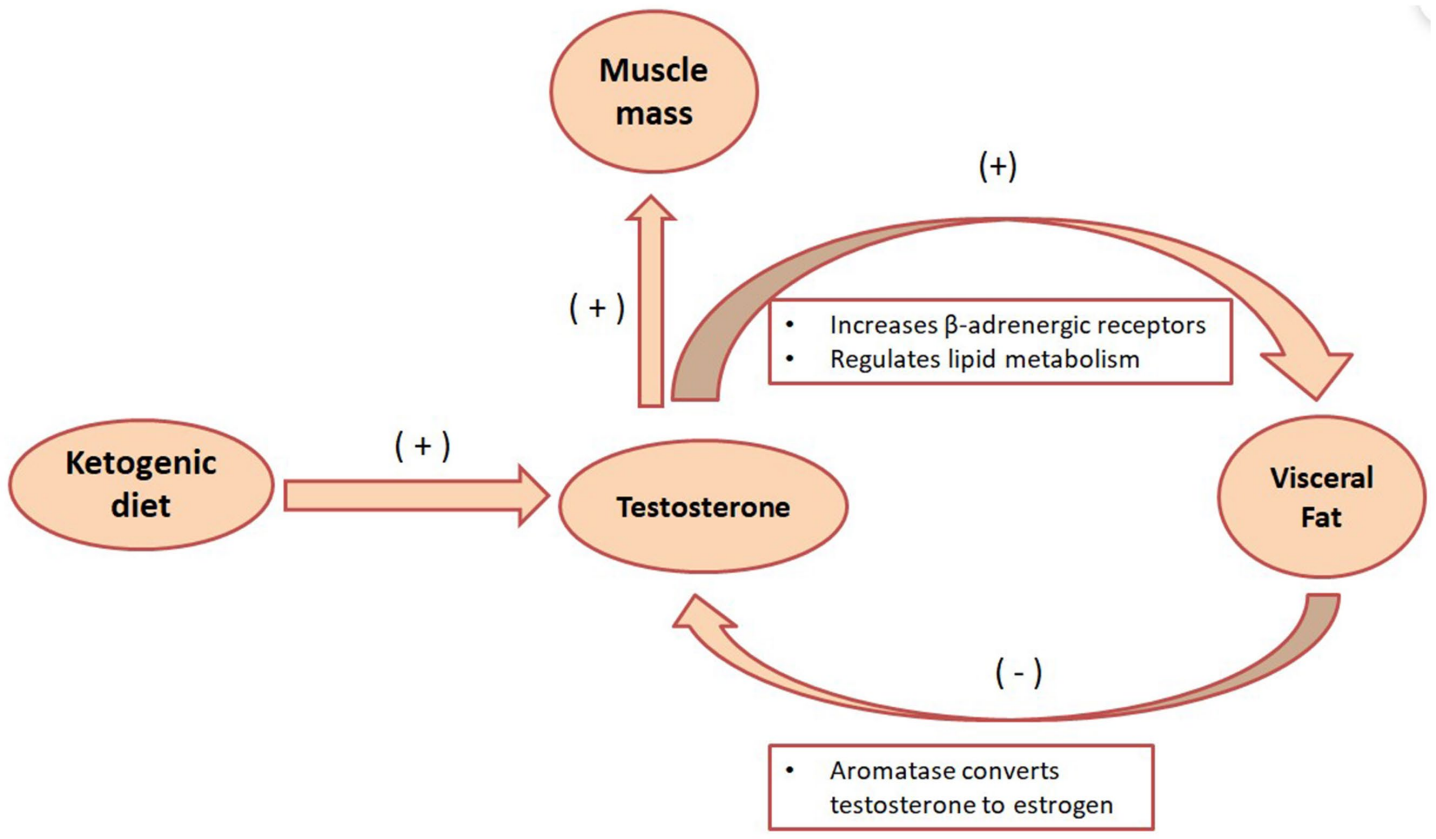
The metabolic role of testosterone in the KD. The KD can increase testosterone secretion, and testosterone can enhance the lipid breakdown effect of norepinephrine by increasing the number of β-adrenergic receptors. Visceral fat also fights testosterone by converting testosterone into estrogen through the action of aromatase.

Visceral fat converts testosterone into estrogen in males through the action of the aromatase enzyme. Consequently, when body fat percentages are elevated, men tend to exhibit increasing estrogen levels, while their muscle-building and fat-burning testosterone concentrations decline, as seen in Figure 2. A recent clinical study presented at the European Endocrinology Conference revealed that the KD benefits overweight males by enhancing testicular hormone profiles and reducing overall obesity markers. After 4 weeks on the KD, 17 male participants showed significant reductions in body weight, fat mass, and BMI. Concurrently, total testosterone and sex hormone-binding globulin (SHBG) levels saw notable increases. As males with obesity or diabetes often suffer from low testosterone and SHBG levels, the data suggests that further investigation into the effects of calorie-restricted KD on male testosterone and SHBG levels is a promising area for additional research (61). Another study divided 20 male participants randomly into two groups: one following a very low-carb KD and the other a low-carb, high-fat, and high-protein diet coupled with strength training. After an 8-week trial period, both groups exhibited significant elevations in baseline and free testosterone levels (62). Moreover, a systematic review and meta-analysis indicate that both conventional KD and extremely low-calorie KD can elevate testosterone levels in men. The beneficial effects on testosterone appear more pronounced in older participants and those who lose more weight post-ketosis (63). Consuming adequate high-quality fats aids in maintaining healthy cholesterol levels, a precursor is essential for testosterone synthesis. Systematic reviews and meta-analyses suggest that, compared to men on high-fat diets, those on low-fat diets experience testosterone level decreases of 10–15%. Especially striking is the 26% reduction in vegan males on low-fat diets (64), suggesting that a KD centered on lipid metabolism may be more beneficial for male fat reduction, enhancing testosterone levels, and promoting lipid metabolism.

Furthermore, appropriate testosterone levels promote muscle repair and the reconstruction of muscle tissue, simplifying muscle growth. From puberty onward, males display evident athletic performance disparities owing to an increase in circulating testosterone concentrations. At any age, males produce up to 30 times more testosterone post-puberty and have 15 times more circulating testosterone than females (65, 66). The substantial sex disparity in circulating testosterone concentrations has a reproducible dose–response relationship with muscle mass, strength, and circulating hemoglobin. This dichotomy largely elucidates the sex differences in muscle mass, strength, and circulating hemoglobin levels, conferring an energy production advantage of at least 8–12% in males (67). It is plausible to hypothesize that under the KD conditions, males might accelerate muscle gain and subsequently hasten fat metabolism due to higher testosterone levels compared to females. Thus, this speculation warrants further comprehensive research.

#### Menstrual cycle

The metabolic response to ketones in females can differ across the various phases of the menstrual cycle. Studies demonstrate that during the luteal phase (days 14–28 post-ovulation), there is a subdued ketone metabolic response, whereas, in the follicular phase (days 1–14 pre-ovulation), this response is more pronounced. Elevated levels of progesterone, predominant in the luteal phase, can impair insulin sensitivity (68), resulting in premenstrual hyperglycemia and augmented insulin secretion. However, this phenomenon might be modulated by other factors, such as the intake of oral contraceptives. Variability in insulin sensitivity and blood glucose levels suggests that ketone concentrations in females may be reduced during specific periods within the menstrual cycle. An enhanced predilection for food and carbohydrates premenstrually in some women can lead to transient weight increments (69). Research postulates that during a natural menstrual cycle, there is a heightened preference for carbohydrates as the primary substrate for oxidative metabolism (70), which might hinder the attainment of ketosis. In summary, the metabolic fluctuations of the menstrual cycle may hinder the achievement and maintenance of ketosis in premenopausal women. Conversely, the absence of such hormonal oscillations in menopause women likely contributes to a more stable metabolic response to ketosis.

### Energy metabolism

Sex differences exist in immediate energy sources in postprandial and resting states. Women are more prone to incorporate postprandial free fatty acids (FFA) into triglycerides, promoting fat storage and using carbohydrates as an immediate energy source. In contrast, men tend to produce energy through plasma FFA oxidation and store carbohydrates as glycogen (71). Thus, women on KD may be more likely to store fat and face greater difficulties in fat mobilization and consumption, although the exact mechanisms of this sexual dimorphism require further study. During aerobic exercise, women are more inclined to use fat oxidation for energy, while men tend to rely more on carbohydrate oxidation to meet exercise-induced energy demands (72, 73). A study investigating fuel metabolism differences between males and females during prolonged endurance exercises (40 ± 70% of VO₂ max) found that men derive more energy from carbohydrate oxidation during physical activity (74). Therefore, compared with KD, women may more easily utilize fat through exercise, but this hypothesis still needs to be confirmed by large-scale clinical studies.

Sex differences also exist in lipid metabolites. A study illustrated a gender-dependent pattern in lipid metabolite levels of lysophosphatidylcholine (lysoPC), phosphatidylcholine (PC), and sphingomyelin (SM) between female and male rats (75). Relative to male rats, PC and lysoPC tend to be significantly elevated in the plasma of female rats. LysoPC is a biologically active, pro-inflammatory lipid produced by pathogenic activity. It can induce hepatocyte stress, cellular damage, and death, leading to inflammation and fibrosis (76). This implies that, to some extent, the lipid metabolism of females in the resting state is more inclined toward inflammatory rather than oxidative responses, indicating that the ketogenic diet may be less suitable for weight loss in women.

### Muscle metabolism

Engaging in muscle training can enhance basal metabolism, making it easier to burn calories. Numerous academic studies have confirmed that the KD may be particularly effective for muscle growth. In a randomized controlled trial involving 20 male participants, 12 switched from their regular diet to the KD, while the remaining 8 continued with their regular diet. After 6 weeks, those in the KD group had gained 2 pounds of muscle, while the control group gained just under 1 pound of muscle (77). However, in a study focusing on the relationship between the KD and muscle in women, 24 female participants were randomly divided into two groups. Twelve underwent a 4-week KD, and the other 12 adopted a 4-week control diet. Using a mixed-model evaluation for treatment efficacy, the study found that the KD might have adverse effects on muscle fatigue in young and healthy women, potentially influencing their sense of fatigue in daily life (78). This trial suggests that the negative impact of the KD on female muscle endurance could be a factor affecting its weight loss efficacy. Perhaps prolonged adaptation to the KD can circumvent these effects. It is crucial to further explore the long-term effects of this diet on muscle fatigue.

### Intestinal structure and gut microbiota

**Figure 3:**
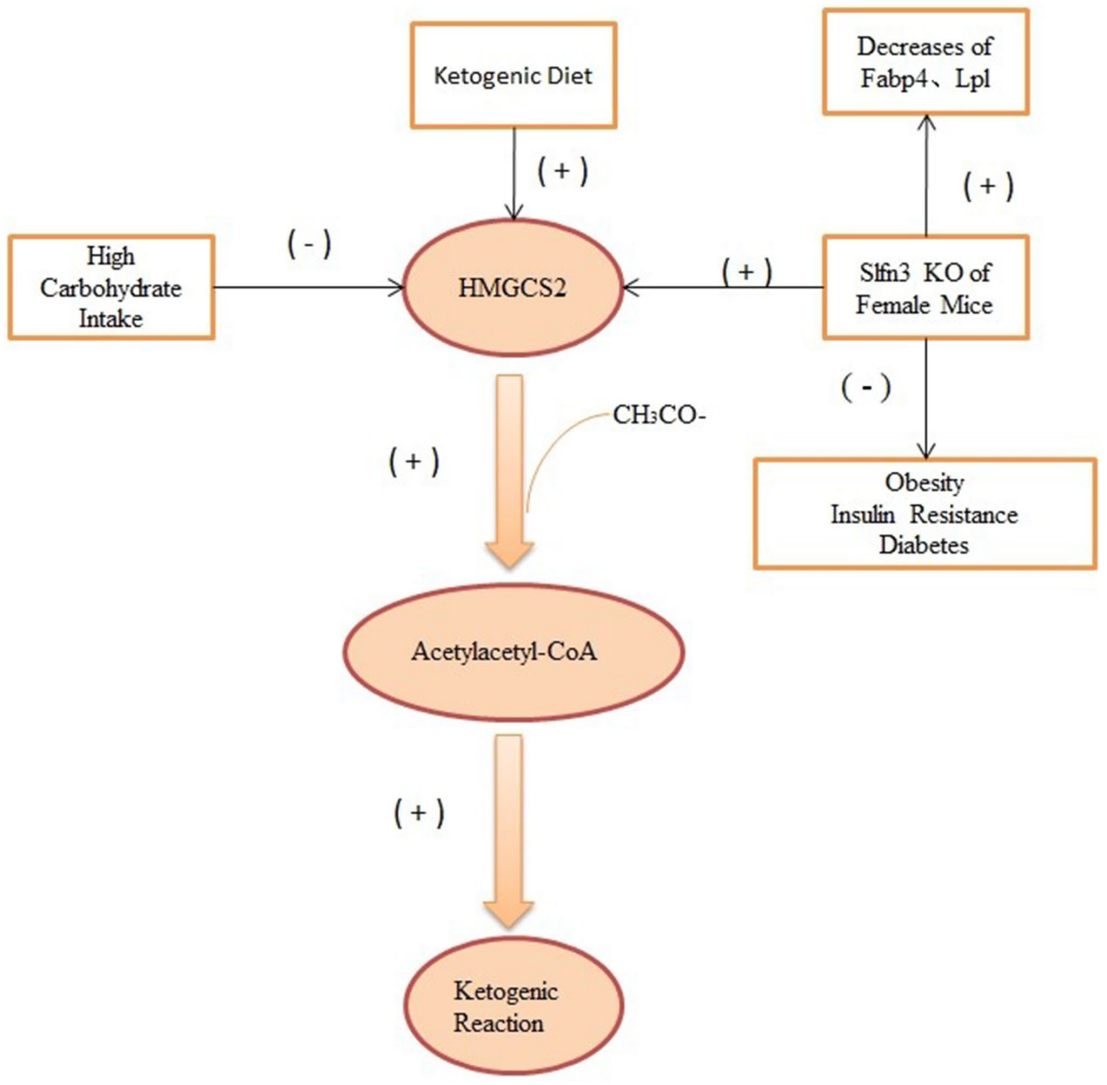
The effect of HMGCS2 on energy metabolism under the KD. HMGCS2 can catalyze ketogenic reaction by acetoacetyl-CoA, while both high-glucose diet and Slfn3 inhibit the expression of this enzyme, thereby inhibiting ketogenic reaction. Elevated HMGCS2 was seen in Slfn3 knockout female mice.

Ketone metabolism refers to the process by which the human body utilizes ketones produced from fat metabolism for energy. Regarding sex differences, studies indicate that there are some disparities between males and females in ketone metabolism. 3-Hydroxy-3-Methylglutaryl-CoA Synthase 2 (HMGCS2) is a rate-limiting enzyme that encodes for the breakdown of FA into ketones, catalyzing the second rate-limiting step in ketogenesis by adding a third acetyl group to acetoacetyl-CoA (79). A high-glucose diet reduces HMGCS2 at the base of the mouse small intestinal crypt and lowers β-OHB levels in the small intestinal crypt (12). Female mice with Slfn3 knockdown showed a more significant reduction in adipogenic genes Fabp4 and Lpl than their male counterparts. Furthermore, the study found sex-specific increases in the ketogenic gene Hmgcs2. Compared to wild-type female mice, female mice with Slfn3 knockdown exhibited a significant increase in Hmgcs2, while no significant change was observed in males (80). The effect of HMGCS2 on energy metabolism under the KD is as shown in Figure 3. As Slfn3 is one of the essential genes regulating intestinal epithelial differentiation, variations in intestinal structure and microbial metabolism might influence weight loss outcomes under ketogenic conditions. However, there is limited research in this domain, warranting further exploration.

The interplay between gut microbiota and adipose distribution showcases distinct sex dichotomies. In a seminal study involving 222 participants, subjects were stratified into four cohorts based on the dichotomy of ‘pear-shaped’ versus ‘apple-shaped’ physiologies and sex distinctions. Astoundingly, certain bacterial strains emanating from the same two genera, “Holdemanella” and “Gemella,” manifested divergent associations with adipose patterning between males and females (81). Within these sex delineations, identical genera could elicit varying correlations with fat distribution, contingent on the constituent bacterial strains. This posits an intriguing query: Under the milieu of the KD, might the microbial interplay exhibit sex-specific nuances? Empirical evidence suggests that the KD might proffer salubrious advantages to those with obesity by orchestrating shifts in the gut microbial landscape, notably by enhancing the Bacteroidetes to Firmicutes ratio and amplifying Prevotella concentrations (82). Throughout one’s ontogeny, concomitant with the maturation of both immune and neural architectures, the evolution of the gut microbiome demonstrates sexual dimorphism, culminating in divergent microbial assemblages in adult males and females. A meticulous denaturing gradient gel electrophoresis (DGGE) scrutiny of the Bacteroides genus revealed an enriched abundance of the polymorphic Bacteroides subtype in males (83). A 4-week ketogenic diet intervention in 17 overweight adults led to a significant reduction in the abundance of Actinobacteria and Firmicutes, while the relative abundance of Bacteroidetes increased (84). These polymorphic Bacteroides, which represent a dominant contingent within the human intestinal milieu, excel at carbohydrate catabolism. They adeptly deconstruct complex plant-derived carbohydrates into glucose and other assimilable saccharides. Given the inherent male predilection to metabolize carbohydrates as a primary energy substrate under specific conditions, and juxtaposing this with the KD’s carbohydrate-sparse nature, this microbial disparity, rooted in carbohydrate metabolism, might potentiate enhance lipid oxidation in males.

The concept of gut microbiota α-diversity encapsulates the heterogeneity within an individual’s microbial communities, denoting the species richness within each assembly. Rodent-centric investigations unveiled a pronouncedly augmented α-diversity in non-obese diabetic female mice during their post-pubertal phase, spanning 10 to 13 weeks, in stark contrast to their male counterparts. Males, however, manifested an elevated prevalence of bacterial families such as Porphyromonadaceae, Peptostreptococcaceae, Lactobacillaceae, and Enterobacteriaceae (85). The Porphyromonadaceae lineage correlates with diminished visceral adiposity and a more salubrious metabolic signature (86). Lactobacilli, with their remarkable capacity to modulate adipocytic mediators, present formidable anti-obesity properties when confronted with a high-fat dietary milieu (87). Notably, studies have emphasized the critical need to prioritize the source (omega-6/omega-3, PUFAs and MUFAs) and quality of fats in KD, as they may differently affect gut microbiota richness and diversity (88). Next-generation sequencing (NGS) or metagenomic sequencing technologies hold promise for enhancing the accuracy of future investigations. These sex differences in gut structure, HMGCS2 enzyme activity, and gut microbiota composition and function could underlie the greater benefits of the ketogenic diet for men. Further research into these factors is warranted.

### Other aspects

Other factors like brain tissue structures and societal elements also play a pivotal role in dictating the differential weight loss responses between men and women. Researchers were able to transform obese male mice into healthier counterparts by curbing appetite and amplifying physical exertion. However, this strategy proved futile in female mice. The cerebral architecture governing caloric utilization showcases sex variances, steered primarily by the neuropeptide pro-opiomelanocortin (POMC) in specific brain regions. POMC peptides in this neural territory are cardinal regulators of appetite, physical activity, energy expenditure, and body weight. Yet, in female mice, the modulatory potency of POMC peptides over physical activity and energy expenditure is not as pronounced (89). Studies revealed that the metabolic glutamate receptor 5 (mGluR5) within steroidogenic factor 1 (SF1) neurons is not quintessential for energy balance regulation. Another investigation illuminated that in the ventromedial hypothalamus of female mice, mGluR5 within SF1 neurons is imperative for glucose homeostasis, whereas this is not the case for males (90). Consequently, when mGluR5 is absent, the neuronal activity of SF1 in female mice is compromised. This derangement flips the protective role of estrogen in glucose metabolism to a detrimental one, impinging on glucose regulation, ushering in glucose intolerance, and exacerbating obesity. Ketone bodies activate the cAMP/CREB pathway, which in turn boosts the expression of brain-derived neurotrophic factor (BDNF). Given that the functionality of mGluR5 in certain brain areas is modulated by BDNF (91), this proffers an explanation as to why males might find it more facile to shed weight on the KD under neuronal impairment conditions. Overall, these neurobiological differences suggest that men may be more suited to the ketogenic diet than women.

## Conclusion and outlook

Overall, obesity manifests differently in men and women, and therefore, the efficacy of the KD in treating obesity is influenced by gender differences. We have summarized the mechanisms underlying gender differences in weight loss induced by the ketogenic diet, as detailed in Table 1. Based on current literature, it can be concluded that KD is most effective for men, followed by postmenopausal women, while its efficacy is most limited in premenopausal women. Compared to men, women exhibit distinct characteristics in fat metabolism under KD: women have lower sensitivity to lipolytic agents such as catecholamines; they face greater challenges in mobilizing and utilizing fat when dietary carbohydrates are reduced; they encounter more difficulties in increasing muscle mass and promoting muscle metabolism; their gut microbiota contains fewer beneficial fat-metabolizing bacteria; their neural regulation of glucose and lipid metabolism is more complex; the menstrual cycle influences their metabolism; and KD may adversely affect muscle fatigue in young, healthy women. Consequently, KD may be more suitable for weight loss in men than in women. These differences may be attributed to factors such as genetics, immunity, gene expression, sex hormones (e.g., testosterone, progesterone, and estrogen), gut microbiota, and neurotransmitters. In summary, this review analyzes the differences in body composition and fat metabolism between the sexes, as well as the resulting variations in KD efficacy. This provides insights for improving existing weight loss strategies, facilitating personalized prevention and treatment measures, and helping to alleviate the public health challenges posed by obesity.

**Table 1:** The summaries of mechanisms about gender differences in weight loss caused by the KD.

Aspects	Male	Female	Reference
Heredity	Catecholamines induce a lipolytic reaction, which is more pronounced in men	The α 2-Adrenaline receptor affinity of women has specificity on different parts on their bodies	(44, 45)
Sex hormone			
Estrogen	/	Higher estrogen levels in women’s bodies suppress sensitivity of α adrenergic receptors to hinder lipolysis, which may lead to insulin resistance	(46–49)
Testosterone	More testosterone in men’s bodies increases muscle synthesis and the number of β adrenergic receptors to promote lipolysis in the ketogenic state	/	(50–60)
Menstrual cycle	/	During the menstrual cycle, women may make it difficult for their bodies to enter the ketogenic state and lose weight.	(61–63)
Energy metabolism	Men may accelerate the mobilization and consumption of fat more easily when going through a decrease in carbohydrates	Lipid metabolites produced by women are more damaging	(64–69)
Muscle metabolism	The KD can increase muscle synthesis in men and improve skeletal muscle mass	The KD may increase muscle fatigue and reduce muscular endurance in women	(70, 71)
Structure and flora of intestinal	Men may have a higher abundance of beneficial flora for fat metabolism	Women may more depend on certain regulatory factors such as SN in intestinal structure than men	(72–79)
Others	Some driving medium such as POMC in the brain of men may play a bigger role on appetite, energy expenditure and body weight	Women have more precise requirements for neuronal regulation of glucose and lipid metabolism	(80–83)

## Limitations

Although a substantial body of literature has elucidated the physiological mechanisms underlying weight loss through the KD, and a considerable number of studies support the existence of sex differences in this phenomenon, several limitations remain. Notably, there is still a lack of long-term follow-up studies, large sample sizes, and high-quality large-scale clinical trials of a diverse population providing direct evidence to substantiate these differences.

Furthermore, research on the interaction between gut microbiota and sex differences in response to KD remains limited, highlighting the need for further exploration in this area. This suggests that sex-related differences in KD effects warrant deeper investigation and may represent a promising avenue for future research.

Nevertheless, there is optimism regarding the development of sex-specific short- and long-term clinical weight loss strategies based on this emerging field. It is also advisable to incorporate sex-stratified analyses when examining the effects of KD on various diseases.

However, it is crucial to acknowledge that most human studies on sex differences are deeply influenced by ethical considerations and have been predominantly conducted in Caucasian populations. The lack of research on sex-related differences in Asian and African populations is evident, underscoring an urgent need for more inclusive and diverse research efforts in the future.

## Glossary

KD: the ketogenic diet
TCA cycle: the tricarboxylic acid cycle
β-OHB: β-hydroxybutyrate
CCK: cholecystokinin
BMI: body mass index
FA: fatty acids
FFA: free fatty acids
TNF: tumor necrosis factor
GR: glucocorticoid receptor
DA: dopamine
5-HT: serotonin
HPA: the hypothalamic–pituitary–adrenal
NE: norepinephrine
SHBG: sex hormone-binding globulin
lysoPC: lys phosphatidylcholine
PC: phosphatidylcholine
SM: sphingomyelin
HMGCS2: 3-hydroxy-3-methylglutaryl-CoA synthase 2
POMC: pro-opiomelanocortin
mGluR5: metabolic glutamate receptor 5
SF1: steroidogenic factor 1
